# Reference values for MRI‐derived psoas and paraspinal muscles and macroscopic fat infiltrations in paraspinal muscles in children

**DOI:** 10.1002/jcsm.13049

**Published:** 2022-07-19

**Authors:** Kacper Marunowski, Dominik Świętoń, Włodzimierz Bzyl, Małgorzata Grzywińska, Piotr Bandosz, Dmitry Khrichenko, Maciej Piskunowicz

**Affiliations:** ^1^ Department of Radiology Medical University of Gdansk Gdansk Poland; ^2^ Faculty of Mathematics, Physics, and Informatics University of Gdansk Gdansk Poland; ^3^ Department of Human Physiology Medical University of Gdansk Gdansk Poland; ^4^ Department of Prevention and Medical Education Medical University of Gdansk Gdansk Poland; ^5^ Division of Body Imaging, Department of Radiology The Children's Hospital of Philadelphia Philadelphia PA USA

**Keywords:** Sarcopenia, Children, Normative values, Psoas and parasipinal muscles, Intramuscular fat

## Abstract

**Background:**

Sarcopenia, defined as loss of skeletal muscle mass, is a novel term associated with adverse outcomes in children. Magnetic Resonance Imaging (MRI) is a safe and precise technique for measuring tissue compartments and is commonly used in most routine paediatric imaging protocols. Currently, there is a lack of MRI‐derived normative data which can help in determining the level of sarcopenia. This study aimed to introduce reference values of total psoas muscle area (tPMA), total paraspinal muscle area (tPSMA), and total macroscopic fat infiltrations of the PSMA (tMFI).

**Methods:**

In this retrospective study, the local database was searched for abdominal and pelvic region MRI studies of children aged from 1 to 18 years (mean age (standard deviation (SD)) of 9.8 (5.5) years) performed in the years 2010–2021. Children with chronic diseases and a history of surgical interventions were excluded from the analysis. Finally, a total of 465 healthy children (*n* = 233 girls, *n* = 232 boys) were enrolled in the study. The values of the tPMA, tPMSA, and tMFI were measured in square centimetres (cm^2^) at the level of the L4/L5 intervertebral disc as the sum of the left and right regions. Age‐specific and sex‐specific muscle, fat, and body mass index percentile charts were constructed using the LMS method. Inter‐observer agreement and intra‐observer reproducibility were assessed using the Bland–Altman plots.

**Results:**

Both tPMA and tPSMA showed continuous increases in size (in cm^2^) throughout all age groups. At the age of 18, the median tPMA areas reached 26.37 cm^2^ in girls and 40.43 cm^2^ in boys. Corresponding tPSMA values were higher, reaching the level of 40.76 cm^2^ in girls and 56.66 cm^2^ in boys. The mean value of tMFI within the paraspinal muscles was 5.0% (SD 3.65%) of their total area in girls and 3.5% (SD 2.25%) in boys with the actual difference between sexes up to 0.96 cm^2^. Excellent intra‐observer reproducibility and inter‐observer agreement were noted. Actual mean differences for tPMA were at the level of 0.43 and 0.39 cm^2^, respectively. Mean bias for tPSMA was 0.1 cm^2^ for inter‐observer and 0.05 cm^2^ for intra‐observer measurements.

**Conclusions:**

Our findings demonstrate novel and highly reproducible sex‐specific MRI‐derived reference values of tPMS, tPSMA, and tMFI at the level of the L4/L5 intervertebral disc for children from 1 to 18 years old, which may guide a clinician in the assessment of sarcopenia, a prognostic outcome marker in children.

## Introduction

Malnutrition is a well‐known phenomenon during the treatment of childhood cancer and multiple acute or chronic diseases.^1^, ^2^, ^3^, ^4^, ^5^ Loss of skeletal muscle mass and strength or exercise capacity (physical performance) in the adult population is called sarcopenia and has been described as a component of malnutrition. In the literature, paediatric sarcopenia is a new term associated with insufficient muscle mass at a given age and is an independent risk factor for surgical complications and neoplasm associated with severe adverse events.^1^, ^2^, ^5^, ^6^, ^7^, ^8^, ^9^

There are many approaches for the initial assessment of muscle mass and nutritional status. The most readily available are anthropometric measures [body mass index (BMI), waist to height ratio (WtHR), mid‐arm circumference, or skinfold thickness] as well as biochemical markers. Although anthropometric measurements are easy to use and less costly, they assess muscle mass indirectly with a high inter‐observer variance which may limit sensitivity for detecting changes in these methods.^10^, ^11^, ^12^, ^13^ A good correlation of muscle mass with biochemical markers has already been proven in a healthy population. However, their values may be imprecise due to disease‐related complications such as organ failure or dietary factors.^13^, ^14^

The need for more precise measurements has resulted in the use of other methods such as bioelectrical impedance absorptiometry (BIA) and dual‐energy X‐ray absorptiometry (DXA). BIA is based on the assumption of the constant chemical composition of a fat‐free‐body.^15^ DXA uses two different energy spectra to differentiate bone and soft tissues based on tissue X‐ray absorption.^16^ However, changes in body composition caused by disease‐related complications such as ascites, organomegaly, organ failure, abnormalities in hydration status, or post‐surgery changes limit their reproducibility in patients with chronic diseases.^10^, ^15^, ^17^

Computed tomography (CT) overcomes these limitations by direct quantification of body compartments. The utility of CT in the assessment of sarcopenia based on a single slice at the abdominal level was already proven both in the adult and paediatric populations.^2^, ^3^, ^4^, ^6^, ^8^, ^18^, ^19^ Recently Eberhard *et al*. published normative values of psoas muscle area at the L3/L4 and L4/L5 intervertebral lumbar levels.^20^ However, due to radiation exposure, the CT examination of abdominal and pelvic levels in children and adolescents is performed primarily during an emergency. Diagnostic and control tests of chronic diseases in these areas are performed using magnetic resonance imagining (MRI). MRI is a radiation‐free technique and is considered the gold standard technique in soft tissues segmentation. It enables the assessment of muscle area as well as the measurement of intramuscular adipose tissue. As a measurement of cross‐selectional muscle area at the L4/L5 intervertebral disc correlates with whole‐body muscle mass and MRI is increasingly available in clinical centres, the evaluation of sarcopenic status can be included in the routine diagnostic protocols of abdominal/pelvis cavity examinations.^8^, ^20^, ^21^ This study, therefore, was performed in a healthy paediatric population to establish the sex‐dependent reference normative values of MRI‐derived psoas and paraspinal muscles area and display a normal range of macroscopic fat infiltration into paraspinal muscles.

## Material and methods

### Patients

This retrospective, cross‐sectional single‐centre study was approved by the Institutional Ethics Committee (NKBBN/443/2018). The sample population included MRI examinations of the abdomen, pelvis, and lumbar spine in children and adolescents aged 0 to 18 years.

The dedicated search engine MedStream Designer (MSD) was used to search the local database for examinations performed in the years 2010–2021 where 2175 records were found. Exclusion criteria consisted of other body regions studies incorrectly found by MSD (*n* = 433), examinations without T2‐weighted sequences in the transverse plane (*n* = 27), T2‐weighted sequences distorted by artefacts (*n* = 6), children with a history of the oncological or haematological disease, ascites, glycogen storage diseases, muscular dystrophies, a chronic inflammatory/connective tissue diseases (*n* = 637), patients post nephrectomy or other surgical procedure (*n* = 92), children with severe scoliosis, bone or spinal canal disorders, and cerebral palsy (*n* = 47). Only the first MRI examination of changes with benign origin (e.g. liver haemangioma) was included while the rest of the follow‐up studies were excluded from the analysis (*n* = 104). Initially found records included 364 examinations of children in the first year of life. Most of them were excluded due to malignancy and vesicoureteral reflux (*n* = 328), and only 16 records were considered healthy infants. Due to insufficient sample size, all data of children during infancy was excluded from further processing.

The final analysis included a total of 465 healthy children and adolescents aged 1 to 18 years (*n* = 233 girls, *n* = 232 boys). The patients' age, weight, and height at the time of MRI examination were obtained from DICOM header tags. Body mass index (BMI) was calculated for each subject by dividing weight in kilograms by the square of the height in meters. The differentiation of the study cohort by sex and age is available in the supplementary material (Supporting Information, *Figure* S8).

### Imaging method

For evaluation of psoas muscle, paraspinal muscle, and paraspinal intramuscular fat areas, one slice at the level of the L4/L5 intervertebral disc was chosen from the standard TSE T2‐weighted sequence in the transverse plane. The images were obtained from different MRI systems: Philips Achieva 3.0 TX (Philips Medical Systems Nederlands, Best, the Netherlands) and two 1.5 T systems, Magnetom Aera and Magnetom Sola (Siemens Healthineers, Erlangen, Germany).

### Muscle and fat tissue quantification

Images of all selected patients were retrieved from the local picture archiving and communication system. The first fully separated intervertebral space above the sacral bone was considered the L5/S1 level. On sagittal and coronal sequence images the L4/L5 intervertebral disc was identified and the corresponding axial image was selected. If the intervertebral disc was displayed on more than one cross‐section, the image closer to the L4 vertebrae body was selected.

The muscle tissue was segmented into psoas (PMA) and paraspinal muscle (PSMA) compartments. The paraspinal compartment included multifidus, iliocostalis lumborum, and longissimus muscles. Both arms and other abdominal muscles were excluded from muscle tissue quantification. Macroscopic fat infiltrations into PSMA (MFI) were identified and excluded from final muscle areas.

Parametric Magnetic Resonance Imaging v1.2.31‐b (pMRI) freeware available at the website www.parametricmri.com was used for semi‐automatic body composition analysis.

The T2‐weighted sequence was loaded into the volumetric region of interest (ROI) analysis module of pMRI software and processed for segmentation and volumetric quantification of selected tissues. Bilateral PMA and PSMA were delineated on corresponding axial cross‐sectional images by the use of the free hand ROI mode. Signal intensity thresholds were manually set for the analysis of MFI. After corresponding free hand and signal intensity‐based segmentation, all data sets were visually revised and misclassified tissues were corrected.

The total PMA (tPMA), PSMA (tPMSA), and MFI (tMFI) represent the sum of the right and left ROIs (in cm^2^). Total muscle area (TMA) indicates the sum of tPMA and tPSMA muscles (in cm^2^).

**Figure 1:**
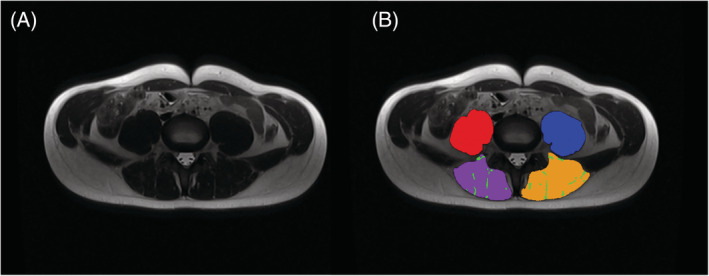
A 17‐year‐old boy (BMI: 19.4 kg/m^2^). (A) Native MRI T2‐weighted sequences in the transverse plane at the level of L4/L5 intervertebral disc. (B) Example of tissue segmentation at the same level: Right PMA (red), left PMA (blue), right PSMA (purple), left PSMA (yellow) and tMFI (green). BMI, body mass index; PMA, psoas muscle area; PSMA, paraspinal muscle area; tMFI, total macroscopic fat infiltrations into PSMA.

Three hundred fifteen sets were segmented by K. M. (3rd year of specialization in radiology) and 150 sets by M. P. (radiologist with 15 years of experience in MRI). Additionally, the average time needed for analysis and correction of a single data set was approximately between 5 and 8 min. The example of the segmented cross‐section is presented in *Figure* 1A,B.

## Statistical analysis

### Reproducibility of image segmentation

The inter‐observer segmentation reproducibility between two independent radiologists (K. M. and M. P.) was assessed based on 40 sets of randomly selected MRI examinations. The evaluation of intra‐observer reproducibility was conducted by re‐segmentation (the time interval to first segmentation was at least 2 weeks) of the same set of images by one of the radiologists (K. M.) Based on these data, intra‐observer and inter‐observer agreement of the segmentation was assessed, using Bland–Altman plots with 95% agreement limits.^22^ Analysis was performed using R, version 4.1.0.

### Statistical analysis of percentile charts

Sex‐specific BMI‐for‐age, tPMA‐for‐age, tPSMA‐for age, TMA‐for‐age and tMFI‐for‐age *z*‐scores and percentile curves were analysed using the LMSChartMaker Light version 2.54 software.^23^ The creation of growth centile standards was based on the lambda‐mu‐sigma method.^24^ The LMS parameters consist of the median (M), the generalized coefficient of variation (S), and the power in the Box‐Cox transformation (L). By the use of Box‐Cox, power transformation measurement for any given age can be brought closest to normality, where age is used as a continuous variable. In the preparation stage, the *z*‐score plot of each variable was inspected for the identification of outliers. None of the outliers were considered to be made due to mistakes in data recording or transferring.

Following WHO guidelines,^25^ derivation of percentiles was enabled only within the interval of *z*‐scores between −3.0 and 3.0. To prevent distortions of percentile charts due to outliers, the data beyond the limits of observed values were fixed at the distance of −2.5 standard deviation (SD) and 2.5 SD at each age correspondingly. Both in boys and girls only two BMI values were fixed. All muscles and tMFI values were within the range of set limits.

The age‐dependent variation of three smooth curves representing L(t), M(t), and S(t) were created from the original data by fitting natural cubic splines with knots at each distinct age t:

$$\mathrm{C\alpha}\left(t\right)=M\left(t\right)\;\times \;{\left[1+L\left(t\right)\;\times \;S\left(t\right)\;\times \;\mathrm{Z\alpha}\right]}^{1/L\left(t\right)}$$

where Zα is the α‐quantile of a standard normal distribution and Cα(t) is a percentile corresponding to Zα. Equivalent degrees of freedom (edf) L(t), M(t), and S(t) measure the complexity of each fitted curve. In our data set, each model was fitted with the loop option. As further fitting made no significant improvements to our models, the standard edf of L3, M5, and S3 was chosen.^23^

## Results

The image segmentation reproducibility analysis showed good agreement for the measurements at the L4/L5 intervertebral level. For tPMA, the mean intra‐observer and inter‐observer disagreements were at the level of 0.43 and 0.39 cm^2^, respectively. On the Bland–Altman intrarater plot, 4 outliners can be spotted, however, most measurements varied up to 1.2 cm^2^. Between independent radiologists, almost all of the actual differences were within the range of 1 cm^2^. The variation and maximal differences of tPSMA actual measurements were slightly higher than tPMA reaching 2 cm^2^ for inter‐observer and to 1.75 cm^2^ for the intra‐observer difference. However, the bias of mean differences was lower with corresponding values of 0.1 and 0.05 cm^2^. The mean tMFI intra‐observer and inter‐observer differences were at levels of 0.77 and 0.91 cm^2^, respectively. On the Bland–Altman plots, a few outliners can be spotted; however, most actual disagreements were within the range of 1.2 cm^2^ for intra‐observer and 1.9 cm^2^ for inter‐observer measurements. The differences between observers in the form of Bland–Altman plots are available in the supplementary material (*Figures* S1–S6).

The BMI‐for‐age, tPMA‐for‐age, tPSMA‐for‐age, TMA‐for‐age, and tMFI‐for‐age percentile charts were constructed based on the 465 MRI examinations of children (233 girls and 232 boys) aged 1 to 18 years (mean age (SD) of 9.8 (5.5) years). For male subjects mean age (SD) was 9.46 (5.31) years. For female subjects mean age (SD) was 10.11 (5.68) years.

**Figure 2:**
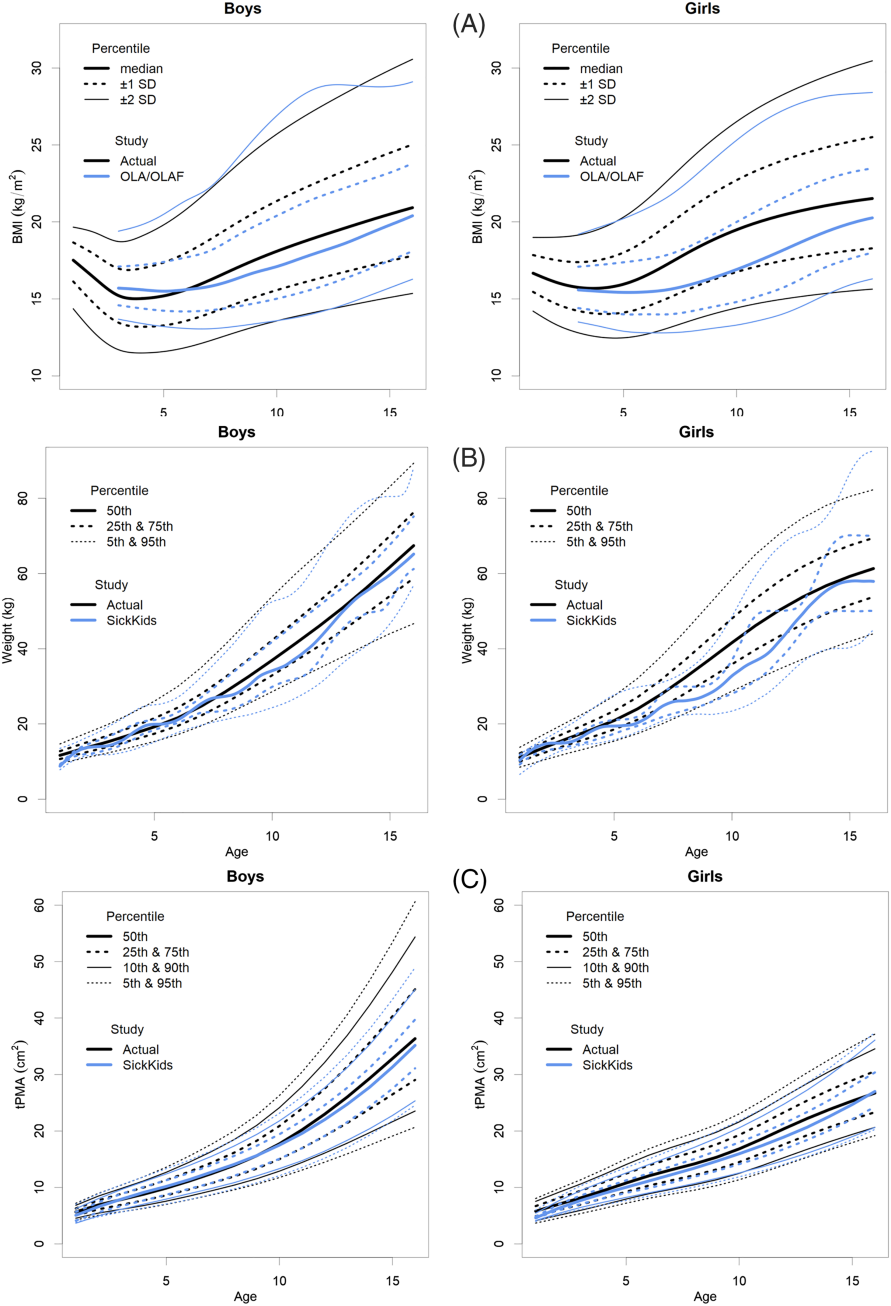
(A) BMI‐for‐age (kg/m^2^) SD percentile charts for boys and girls aged from 1 to 16 years of actual study in comparison with national reference standards (OLA/OLAF study).^26^, ^27^ BMI, body mass index; SD, standard deviation. (B) Weight‐for‐age (kg) percentile charts for boys and girls aged from 1 to 16 years in comparison with weight reference standards published by Eberhard *et al*.^17^ (C) MRI‐derived total‐psoas‐for‐age (cm^2^) percentile charts for boys and girls aged from 1 to 16 years in comparison with tPMA CT‐derived reference standards published by Eberhard *et al*.^17^ tPMA, total psoas muscle area.

The current charts of BMI increase in correlation with age, including SD curves (‐2SD, ‐1SD, median, +1SD, +2SD) and a comparison with the largest BMI study of the Polish population, “Ola/Olaf” are shown in *Figure* 2A.^26^, ^27^ The present study found that the BMI *z*‐scores were higher compared with the national Ola/Olaf reference values, especially in the range of +2 SD and +1 SD, where the differences reached 2.7 units in girls and 1.46 units in boys. As the study population of currently available CT‐derived norms for total psoas muscle area is defined by weight,^20^ the comparison of weights with our results is presented in *Figure* 2B.

**Figure 3:**
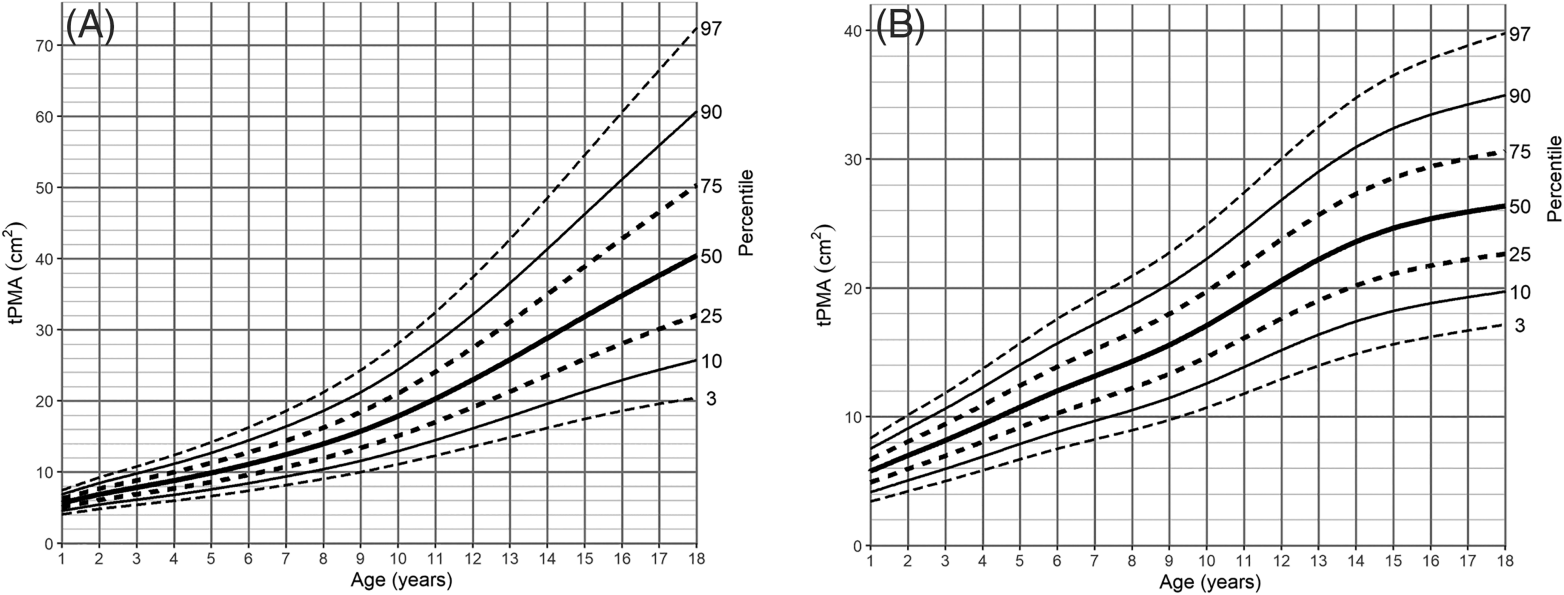
Psoas‐for‐age (cm^2^) percentile charts for (A) boys and (B) girls aged from 1 to 18 years. tPMA, total psoas muscle area.

**Figure 4:**
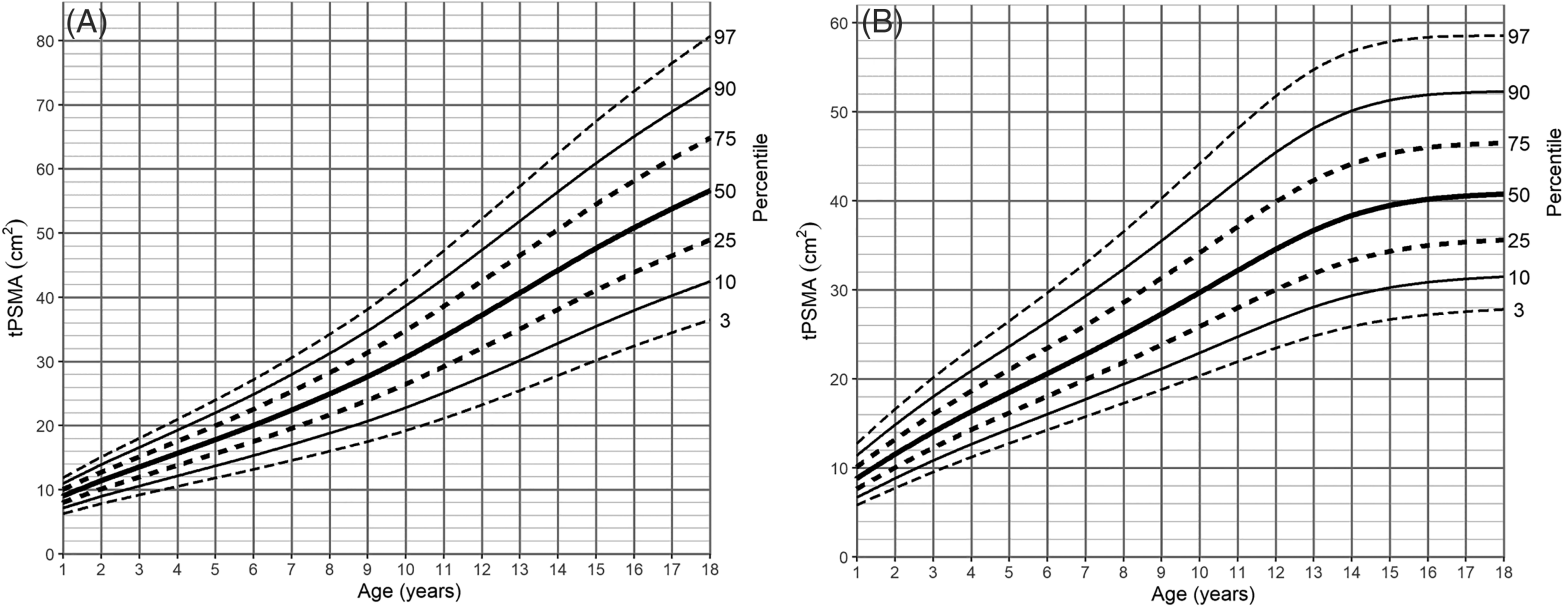
Paraspinal muscle‐for‐age (cm^2^) percentile charts for (A) boys and (B) girls aged from 1 to 18 years. tPSMA, total paraspinal muscle area.

The tPMA and tPSMA reference values for boys and girls in each age group are presented in *Tables* S1 and S2. Corresponding data for TMA is included in *Table* S3. Based on the results, percentile charts were created for each muscle compartment (*Figures* 3A,B, 4A,B, and S7A,B). In all age groups, there was a continuous increase in both tPMA and tPSMA. The sex percentiles values were similar in the first 8 years of life for tPMA and the first 10 years of life for tPSMA, with a later predominance of boys in the following years. In girls, the most dynamic increase in tPMA area was recorded between the ages of 9 and 14, reaching a median of 26.37 cm^2^ at the age of 18. In boys, a dynamic increase of tPMA was observed until the end of the observed range. At this age, a median of 40.43 cm^2^ was reached. The sex distribution of tPSMA percentiles was different in children 14 years of age and older. In boys, an increase was noted within all age groups, while in girls, stability was reached around the age of 15 years. The medians of tPSMA surface at the end of the observed age reached values of 56.66 and 40.76 cm^2^, respectively.

**Figure 5:**
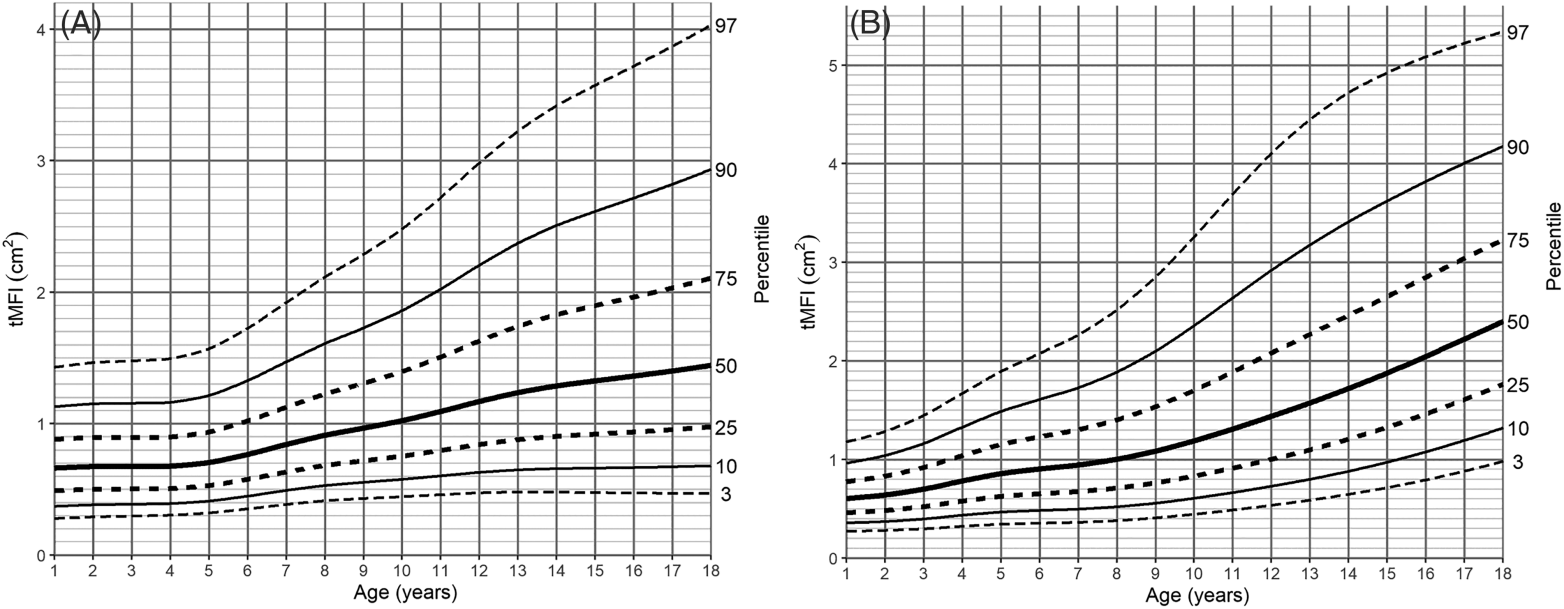
tMFI‐for‐age (cm^2^) percentile charts for (A) boys and (B) girls aged from 1 to 18 years. tMFI, total macroscopic fat infiltrations in paraspinal muscles.

No substantial differences between sexes were noted in the surface area of tMFI from age 1 to 3 years old. In older children, female predominance was observed, especially prominent during adolescence (for medians, the difference of 0.34–0.96 cm^2^). The mean value of tMFI reached up to 5% (SD 3.65%) in girls and 3.5% (SD 2.25%) in boys. The distribution of percentile charts in our population showed a continuous increase observed across all age groups with a steep increase in girls from the age of 8 years onwards (*Table* S4, *Figure* 5A,B).

## Discussion

This study proposed sex‐specific MRI‐derived reference values as percentile charts of the psoas muscle, paraspinal muscles, and macroscopic fat infiltrations in paraspinal muscles areas for children aged from 1 to 18 years. This is the first study that demonstrates normative values of paraspinal muscle and macroscopic fat infiltrations in paraspinal muscles included in the whole single‐slice muscle area. Until now, intramuscular fat of children's paraspinal muscles was only described as circular ROI fat fraction.^28^

With skeletal muscle mass increasing during growth, many pathological states such as malignancies, post‐surgery, or inflammatory diseases may lead to inadequate muscle mass for a given age.^1^, ^2^, ^14^ This state, called sarcopenia, is associated with adverse health outcomes during treatment and a decrease of life quality, mainly described in the adult population.^14^, ^18^, ^19^, ^29^ However, this phenomenon has been described more and more recently in paediatric populations.^2^, ^5^, ^6^, ^7^, ^9^

Currently available methods of measuring sarcopenia are often implicated in disease‐related conditions and appear to be inadequate to meet the demands of personalized medicine that is easily incorporated into clinical practice.^30^

Radiological direct measurements of cross‐sectional muscle area at the lumbar level are proven objective and feasible methods to assess sarcopenia.^3^, ^5^, ^6^ In a recently published study, the reference values for total psoas muscle area were established in a group of children 1–16 years old who required emergency abdominal CT examination after paediatric trauma.^20^ However, most malnutrition abnormalities leading to sarcopenia are diagnosed with MRI. Unlike CT, this imaging method is a preferred part of many paediatric diagnostic protocols, free from ionizing radiation, with much better ‘soft tissue contrast’ than CT.

The reproducibility of MRI examination of tPMA was already demonstrated in the literature.^5^, ^31^ Excellent intra‐observer and inter‐observer agreements were observed in our study (*Figures* S1–S4). The mean disagreement of tPMA surface area at the level of the L4/L5 intervertebral disc obtained by the use of a semi‐automatic method was higher than tPSMA by 0.33 cm^2^ in intra‐observer and by 0.34 cm^2^ in the inter‐observer group. However, actual maximal differences were higher in the group of paraspinal muscles. It indicates a greater variety of measurements with a similar spread on both sides of the middle line. In both muscle groups, the reader (M. P.) selected slightly larger muscle areas than the second reader (K. M.), but the difference was insignificant. The high inter‐observer agreement obtained in our study indicates the feasibility of tissue area percentile charts based on our results.

Accurate and reproducible measurement of adipose and muscle areas based on MRI examinations requires the involvement of personnel experienced with this method. The assessment of full muscle length is time‐consuming and often impossible due to the limitation of the area included in the examination. Moreover, adding dedicated sequences to fully appreciate selected muscles will increase the time of examination and cost, as well as sedation time in younger children.

Following previous studies, estimation of muscle mass based on a single cross‐section is highly accurate; therefore, such an approach is currently the most cost and time‐efficient option.^21^, ^29^, ^32^, ^33^ In the paediatric population, the utility of manual measurement was already proven for tPMA and tPSMA.^2^, ^3^, ^4^, ^6^ Additionally, previous studies noted a continuous increase of both tPMA and tPSMA during the first 20 years of life.^34^ In comparison, accurate quantification of smaller muscles (e.g. abdominal oblique muscles) is possible only when bordering tissues have different signal intensity such as fat. The abdominal wall in the youngest and thinnest children contains little adipose tissue, which limits the proper identification of muscle margins.

In the published articles, few paediatric measurement sites were proposed, with the level of the third lumbar vertebrae being most frequently used.^1^, ^2^, ^5^, ^6^, ^8^ Recent studies introduced the level of the L4/L5 intervertebral disc as a feasible cross‐section for single‐slice measurements in children.^3^, ^20^ Lurz *et al*.^20^ proved the interchangeability of L3/L4 and L4/L5 intervertebral disc levels for tPMA measurement in both sexes (*r* = 0.95–0.98, *P* < 0.001–0.01). According to authors, the tPMA differs depending on the measured level with the largest area noted at the level of the upper L5 vertebrae endplate. In comparison, the largest tPSMA was reported at the level of the upper L3 vertebrae endplate, whereas the area was slightly smaller at levels of L4 and L5 vertebrae, but not significant.^34^ In clinical settings, paediatric MRI examination of both pelvis and abdominal region usually covers the L4/L5 level which allows utilization of percentile charts in a greater variety of pathological conditions. Therefore, we had chosen this region as a reference level in our study.

The results of BMI SD scores for children from 1 to 4 years old present a similar configuration to WHO normative values.^25^, ^35^ The distribution of BMI percentile lines of older children in comparison with national Ola/Olaf BMI standards was similar, however, our results for the majority of the time were higher^26^, ^27^ (*Figure* 2A). This was particularly observed for girls from the fifth year of life onwards. The disagreement may be related to our patient study population which included only the Caucasian race. On the other hand, an increase in mean weight and BMI was reported over the years.^36^ The WHO and Ola/Olaf BMI data represent standards from before the year 2010 which may create a slight bias in comparison with the current weight distribution. Bodyweight percentile lines also showed a noticeable difference compared with the Canadian study, while the children included in our study were heavier^20^ (*Figure* 2B).

A further interesting observation in our study is a noticeable difference between sexes for both tPMA and tPSMA percentile lines. In the early years of life, the differences in muscle areas between boys and girls are small; however, from the age of 8 for tPMA and the age of 10 for tPSMA, a greater difference can be noticed in boys. At the end of observed age, flattening of both muscle percentile curves in girls is noticeable, which may be associated with an earlier end of growth phase than in boys. At the end of the observed age range, the slope of percentile lines of tPMA in our study is similar to previously published norms in CT^20^ (*Figure* 2C). When compared with the Canadian study,^20^ our 75th, 90th, and 95th tPMA percentile lines tend to have a little bit greater differences in older children. It may be the result of an insufficient sample size and also a greater variance of results of enrolled children. However, considering the difference in populations, tPMA growth tendencies between both studies showed high convergence overall.

Measurement of muscle area in the paediatric population was already conducted by the use of both T1‐weighted and T2‐weighted sequences,^5^, ^31^ while MFI was only evaluated in T1‐weighted water‐fat sequences (called Dixon sequence).^28^ T1‐weighted Dixon sequence is characterized by short acquisition time, however, is vulnerable to movement and breathing artefacts, which occur much more often in children than adults especially if children are sedated. Hence, for MFI evaluation, we decided to use regular T2‐weighted sequences which are part of standard abdominal and pelvis examination and their sensitivity and specificity in the assessment of adipose tissue are comparable with T1‐weighted sequences.^37^ During adolescence and early adulthood, greater quantities of intramuscular fat were observed in girls than boys, which is compatible with higher fat content in females. The mean value of tMFI in our study (3.5% in boys and 5% in girls) is consistent with available data.^38^ As body fat fraction increases during chronic diseases, malignancies, and treatment with specific drugs (e.g. steroids),^39^ area‐for‐age normative values may be used to estimate the degree of fat infiltration. In those cases, it is especially important to measure fat‐free muscle area as the inclusion of MFI may delay the diagnosis of sarcopenia. Additionally, it was reported that the functionality of lower extremity muscles seems to be disturbed by fat infiltrations.^40^ We suspect that similar changes occur in paraspinal muscles, which alongside muscle loss in sarcopenia may further decrease physical performance. It indicates that the assessment of MFI may become part of the initial evaluation during hospitalization in the future.

This study also has several limitations. Firstly, our study was performed at a single institution; therefore, our results only refer to the Caucasian population. Secondly, there is a lack of percentile charts for children in the first year of life. It was caused by an insufficient number of healthy participants because most MRI examinations of the abdomen and pelvis were performed for oncological reasons. Further, the tPMA, tPSMA, and tMFI were not standardized by height, which limits the consistency of measurements across the range of growing deficits. However, height in children depends on both constitutional and health‐related variables. Growth deficits and sarcopenia are often related to the same underlying conditions. Height‐standardization, may improve the performance of charts but also introduce overcorrection, especially in long‐lasting diseases which are affecting the subject's height. Currently, there is a lack of MRI‐derived reference values in the paediatric population, and in CT‐based percentile charts height correction was not performed.^20^ Additionally, the distribution of BMI charts in our group was similar to the national growth charts which reflect the general population of Poland^26^, ^27^ (*Figure* 2A). Therefore, no correction for height was performed, and we accepted the methodology consistent with previous studies.^20^ Instead, we rather recommend the clinical use of percentile charts taking into account the broader clinical context. The last limitation includes the inability to estimate confidence intervals for each percentile line during the construction of charts and the time necessary to perform semi‐automatic segmentation of tissues. Multicentre studies on larger populations may require the implementation of fully automatic tools such as currently rapidly developing deep learning algorithms.

In conclusion, our study is the first to define MRI‐derived reference values of tPMA and tPSMA in the form of percentile charts for children aged from 1 to 18 years. Additionally, tMFI was measured and first age‐dependent norms for boys and girls during childhood and adolescence were presented. In the view of personalized medicine, early detection of sarcopenia and macroscopic fat infiltrations into paraspinal muscles seems to be one of the important directions in paediatric medicine. Incorporation of our method into standard diagnostic protocols may enable a precise initial assessment of sarcopenic status and appropriate nutritional support.

## Conflict of interest

Kacper Marunowski, Dominik Świętoń, Włodzimierz Bzyl, Małgorzata Grzywińska, Piotr Bandosz, Dmitry Khrichenko, and Maciej Piskunowicz declare that they have no conflict of interest.

## Funding

This study received no specific grant from any funding agency in the public, commercial, or not‐for‐profit sectors.

## Supporting information


**Figure S1.** Bland–Altman plot of the difference in interobserver tPMA segmentation (cm^2^) against the mean tPMA segmentation (cm^2^). KM, first radiologist; MP, second radiologist; tPMA, total Psoas Muscle Area; SD, standard deviation.

**Figure S2.** Bland–Altman plot of the difference in interobserver tPSMA segmentation (cm^2^) against the mean tPSMA segmentation (cm^2^). KM, first radiologist; MP, second radiologist; tPSMA, total Paraspinal Muscle Area; SD, standard deviation.

**Figure S3.** Bland–Altman plot of the difference in interobserver tMFI segmentation (cm^2^) against the mean tMFI segmentation (cm^2^). KM, first radiologist; MP, second radiologist; tMFI, total Macroscopic Fat Infiltrations of the paraspinal muscle area; SD, standard deviation.

**Figure S4.** Bland–Altman plot of the difference in intraobserver tPMA segmentation (cm^2^) against the mean tPMA segmentation (cm^2^). KM_1_, first radiologist, first measurement; KM_2_, first radiologist, second measurement; tPMA, total Psoas Muscle Area; SD, standard deviation.

**Figure S5.** Bland–Altman plot of the difference in intraobserver tPSMA segmentation (cm^2^) against the mean tPSMA segmentation (cm^2^). KM_1_, first radiologist, first measurement; KM_2_, first radiologist, second measurement; tPSMA, total Paraspinal Muscle Area; SD, standard deviation.

**Figure S6.** Bland–Altman plot of the difference in intraobserver tMFI segmentation (cm^2^) against the mean tMFI segmentation (cm^2^). KM_1_, first radiologist, first measurement; KM_2_, first radiologist, second measurement; tMFI, total Macroscopic Fat Infiltrations of the paraspinal muscle area; SD, standard deviation.

**Figure S7.** Total muscle area‐for‐age (cm^2^) percentile charts for a. boys and b. girls aged from 1 to 18 years. TMA, Total Muscle Area.

**Figure S8.** Study cohort differentiated by sex and age.

**Table S1.** tPMA‐for‐age (cm2) references for boys and girls. SD, standard deviation; tPMA, total Psoas Muscle Area

**Table S2.** tPSMA‐for‐age (cm2) references for boys and girls. SD, standard deviation; tPSMA, total Paraspinal Muscle Area

**Table S3.** TMA‐for‐age (cm2) references for boys and girls. SD, standard deviation; TMA, Total Muscle Area

**Table S4.** tMFI‐for‐age (cm2) references for boys and girls. SD, standard deviation; tMFI, total Macroscopic Fat Infiltrations in paraspinal muscles
