# Supplementary figures and images for: Reference values for MRI‐derived psoas and paraspinal muscles and macroscopic fat infiltrations in paraspinal muscles in children
Source: J Cachexia Sarcopenia Muscle. 2022 Jul 19;13(5):2515–24. doi: 10.1002/jcsm.13049 (PMC9530503; doi:10.1002/jcsm.13049)

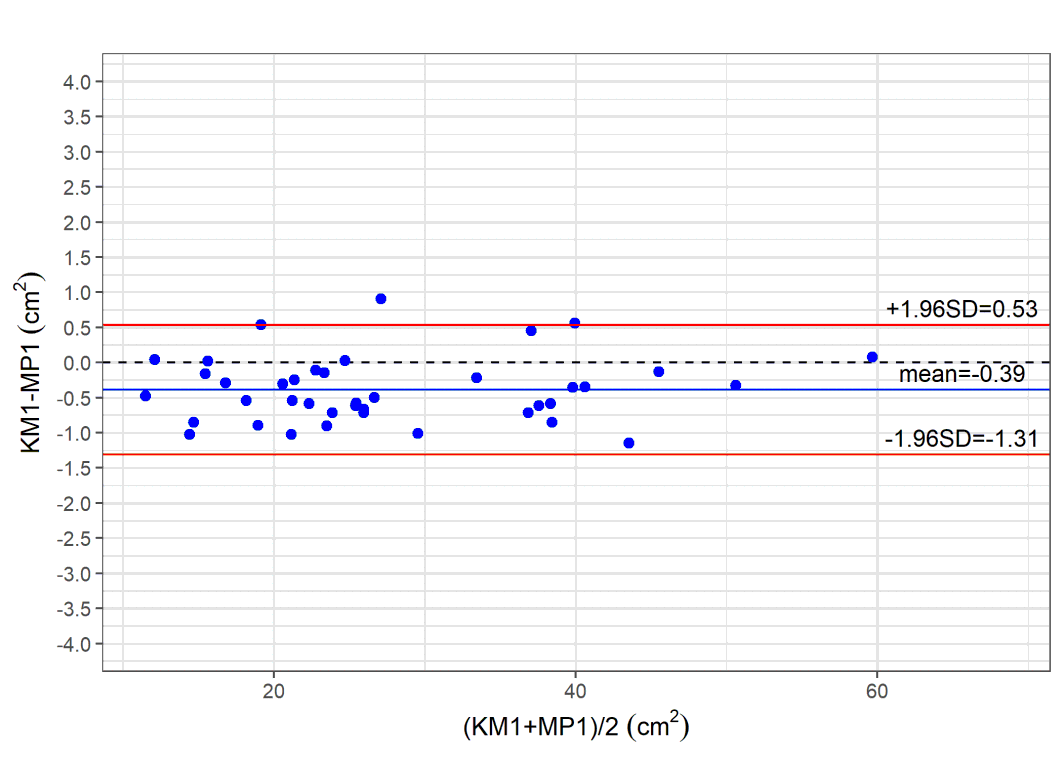

Supplement: Supplementary file 1 — Figure S1. Bland–Altman plot of the difference in interobserver tPMA segmentation (cm2) against the mean tPMA segmentation (cm2). KM, first radiologist; MP, second radiologist; tPMA, total Psoas Muscle Area; SD, standard deviation. [file JCSM-13-2515-s001.tif]

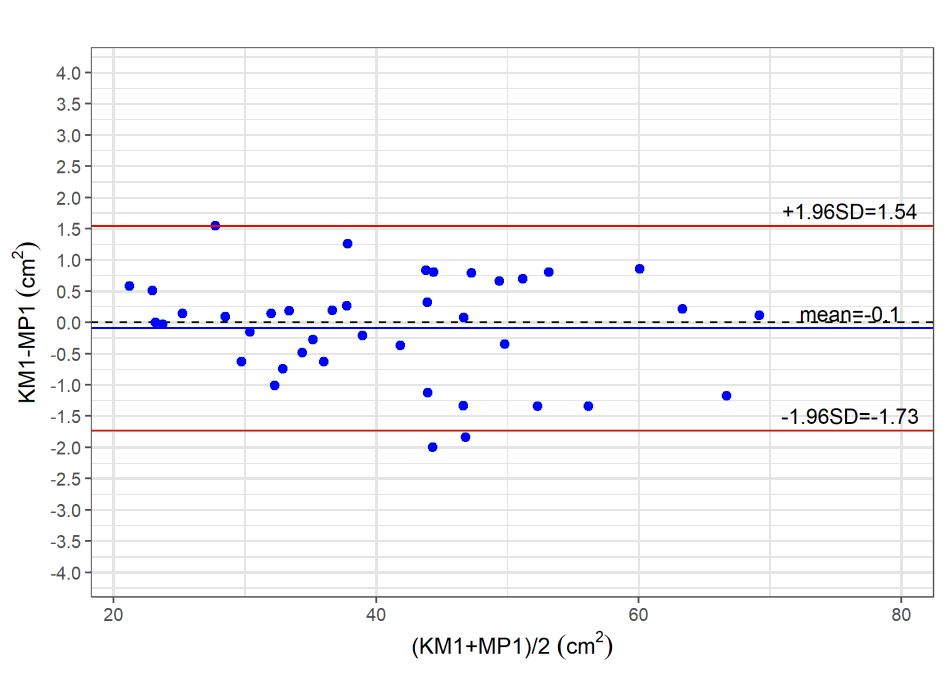

Supplement: Supplementary file 2 — Figure S2. Bland–Altman plot of the difference in interobserver tPSMA segmentation (cm2) against the mean tPSMA segmentation (cm2). KM, first radiologist; MP, second radiologist; tPSMA, total Paraspinal Muscle Area; SD, standard deviation. [file JCSM-13-2515-s008.tif]

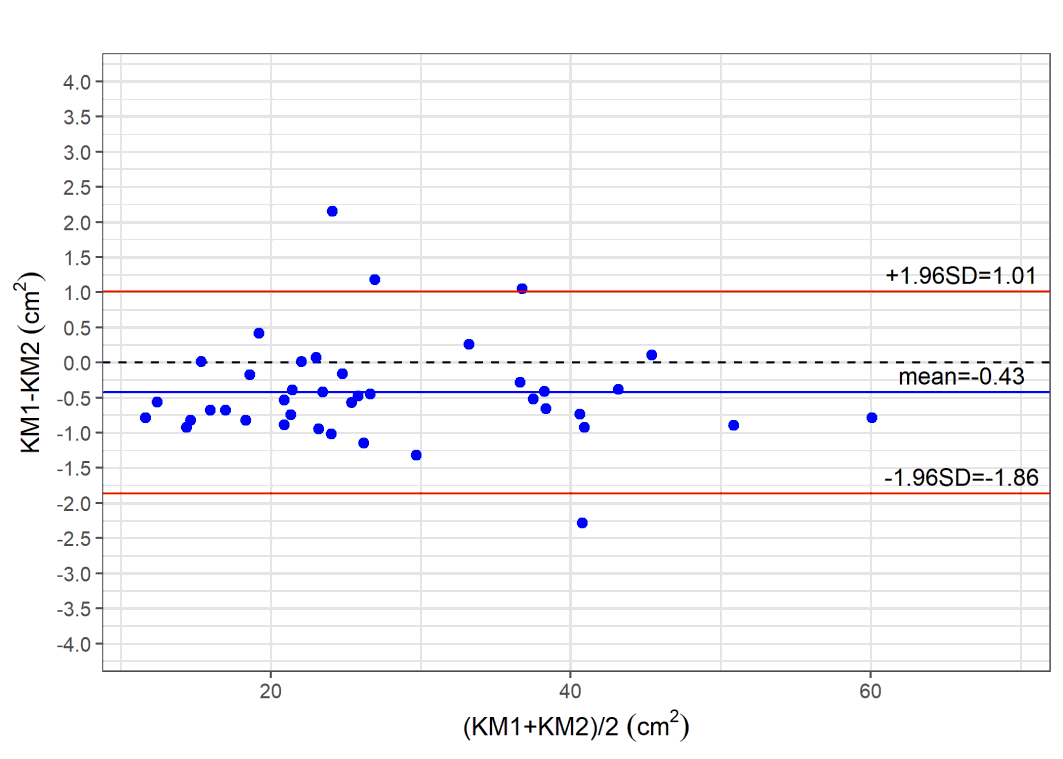

Supplement: Supplementary file 4 — Figure S4. Bland–Altman plot of the difference in intraobserver tPMA segmentation (cm2) against the mean tPMA segmentation (cm2). KM1, first radiologist, first measurement; KM2, first radiologist, second measurement; tPMA, total Psoas Muscle Area; SD, standard deviation. [file JCSM-13-2515-s007.tif]

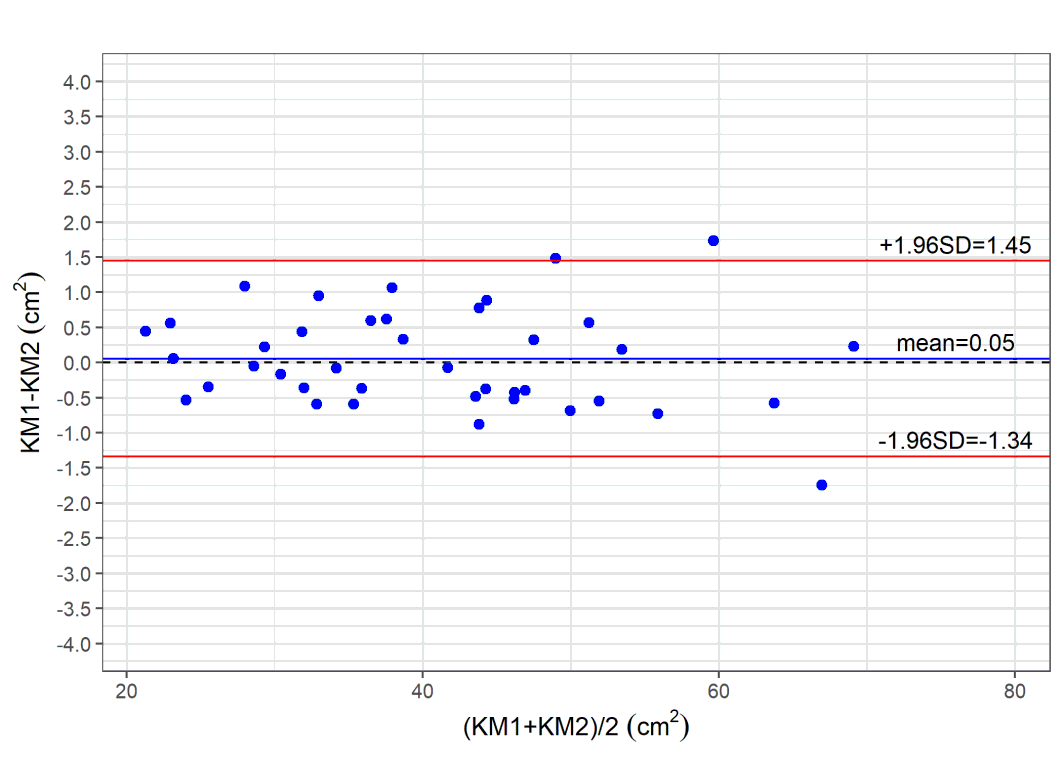

Supplement: Supplementary file 5 — Figure S5. Bland–Altman plot of the difference in intraobserver tPSMA segmentation (cm2) against the mean tPSMA segmentation (cm2). KM1, first radiologist, first measurement; KM2, first radiologist, second measurement; tPSMA, total Paraspinal Muscle Area; SD, standard deviation. [file JCSM-13-2515-s004.tif]

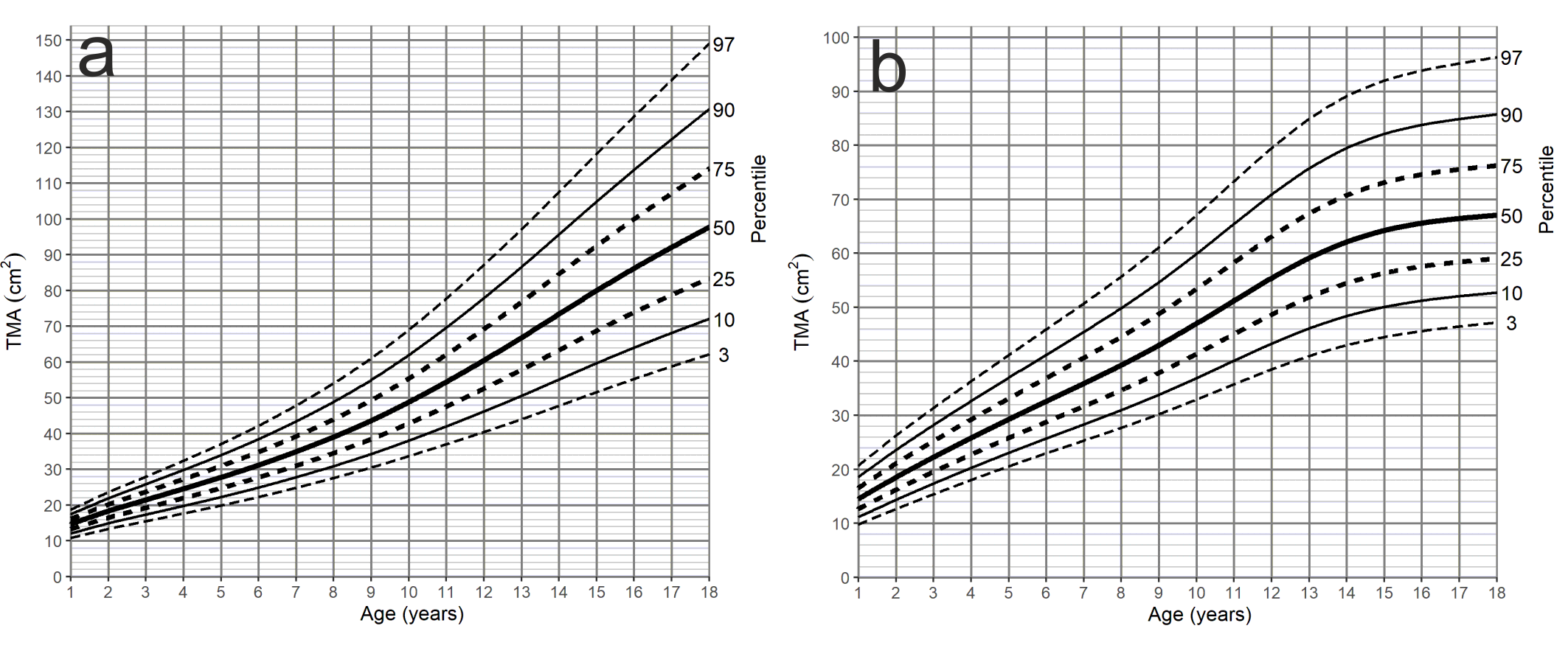

Supplement: Supplementary file 7 — Figure S7. Total muscle area‐for‐age (cm2) percentile charts for a. boys and b. girls aged from 1 to 18 years. TMA, Total Muscle Area. [file JCSM-13-2515-s006.tif]

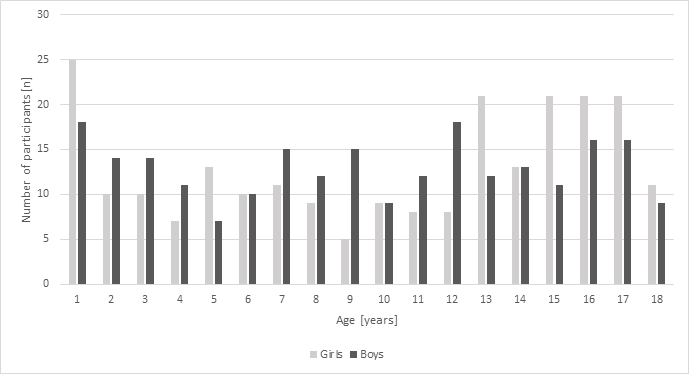

Supplement: Supplementary file 8 — Figure S8. Study cohort differentiated by sex and age. [file JCSM-13-2515-s002.tif]
