# Supplementary material for: Reference values for MRI‐derived psoas and paraspinal muscles and macroscopic fat infiltrations in paraspinal muscles in children
Source: J Cachexia Sarcopenia Muscle. 2022 Jul 19;13(5):2515–24. doi: 10.1002/jcsm.13049 (PMC9530503; doi:10.1002/jcsm.13049)
Supplement: Supplementary file 9 — Table S1. tPMA‐for‐age (cm2) references for boys and girls. SD, standard deviation; tPMA, total Psoas Muscle Area [file JCSM-13-2515-s009.docx]

| **Age (years)** | **Boys** | | | | | | | | | **Girls** | | | | | | | | |  |
| --- | --- | --- | --- | --- | --- | --- | --- | --- | --- | --- | --- | --- | --- | --- | --- | --- | --- | --- | --- |
|  | **-2SD** | | **-1SD** | | **Median** | **1SD** | | **2SD** | | **-2SD** | | **-1SD** | | **Median** | **1SD** | | **2SD** | | |
| 1 | 3.96 | 4.79 | | 5.67 | | | 6.60 | | 7.57 | 3.27 | 4.50 | | 5.79 | | | 7.13 | | 8.52 |  |
| 2 | 4.69 | 5.74 | | 6.88 | | | 8.10 | | 9.41 | 4.05 | 5.47 | | 7.00 | | | 8.62 | | 10.33 |  |
| 3 | 5.24 | 6.47 | | 7.84 | | | 9.35 | | 11.00 | 4.82 | 6.42 | | 8.17 | | | 10.07 | | 12.11 |  |
| 4 | 5.80 | 7.20 | | 8.80 | | | 10.61 | | 12.64 | 5.63 | 7.43 | | 9.43 | | | 11.63 | | 14.02 |  |
| 5 | 6.44 | 8.03 | | 9.89 | | | 12.04 | | 14.51 | 6.47 | 8.48 | | 10.75 | | | 13.27 | | 16.04 |  |
| 6 | 7.16 | 8.97 | | 11.12 | | | 13.66 | | 16.66 | 7.26 | 9.48 | | 12.01 | | | 14.84 | | 17.98 |  |
| 7 | 7.95 | 9.99 | | 12.47 | | | 15.46 | | 19.05 | 7.97 | 10.40 | | 13.16 | | | 16.27 | | 19.71 |  |
| 8 | 8.77 | 11.10 | | 13.97 | | | 17.47 | | 21.73 | 8.65 | 11.30 | | 14.30 | | | 17.66 | | 21.37 |  |
| 9 | 9.69 | 12.39 | | 15.73 | | | 19.86 | | 24.94 | 9.43 | 12.32 | | 15.58 | | | 19.22 | | 23.23 |  |
| 10 | 10.75 | 13.91 | | 17.86 | | | 22.77 | | 28.83 | 10.35 | 13.53 | | 17.11 | | | 21.08 | | 25.44 |  |
| 11 | 11.92 | 15.62 | | 20.30 | | | 26.14 | | 33.38 | 11.39 | 14.89 | | 18.82 | | | 23.19 | | 27.98 |  |
| 12 | 13.14 | 17.48 | | 22.99 | | | 29.90 | | 38.50 | 12.47 | 16.28 | | 20.59 | | | 25.38 | | 30.66 |  |
| 13 | 14.36 | 19.42 | | 25.86 | | | 33.97 | | 44.08 | 13.49 | 17.57 | | 22.22 | | | 27.44 | | 33.23 |  |
| 14 | 15.61 | 21.43 | | 28.89 | | | 38.33 | | 50.13 | 14.39 | 18.66 | | 23.59 | | | 29.20 | | 35.50 |  |
| 15 | 16.80 | 23.40 | | 31.92 | | | 42.77 | | 56.40 | 15.12 | 19.51 | | 24.64 | | | 30.56 | | 37.32 |  |
| 16 | 17.86 | 25.25 | | 34.87 | | | 47.17 | | 62.67 | 15.70 | 20.13 | | 25.38 | | | 31.54 | | 38.68 |  |
| 17 | 18.75 | 26.96 | | 37.70 | | | 51.47 | | 68.80 | 16.20 | 20.62 | | 25.93 | | | 32.27 | | 39.79 |  |
| 18 | 19.49 | 28.56 | | 40.45 | | | 55.70 | | 74.88 | 16.68 | 21.05 | | 26.40 | | | 32.92 | | 40.83 |  |

**Supplementary table 1.** tPMA-for-age (cm^2^) references for boys and girls. SD, standard deviation; tPMA, total Psoas Muscle Area
