# Supplementary material for: Reference values for MRI‐derived psoas and paraspinal muscles and macroscopic fat infiltrations in paraspinal muscles in children
Source: J Cachexia Sarcopenia Muscle. 2022 Jul 19;13(5):2515–24. doi: 10.1002/jcsm.13049 (PMC9530503; doi:10.1002/jcsm.13049)
Supplement: Supplementary file 10 — Table S2. tPSMA‐for‐age (cm2) references for boys and girls. SD, standard deviation; tPSMA, total Paraspinal Muscle Area [file JCSM-13-2515-s010.docx]

| **Age (years)** | **Boys** | | | | | | | | | **Girls** | | | | | | | | |  |
| --- | --- | --- | --- | --- | --- | --- | --- | --- | --- | --- | --- | --- | --- | --- | --- | --- | --- | --- | --- |
|  | **-2SD** | | **-1SD** | | **Median** | **1SD** | | **2SD** | | **-2SD** | | **-1SD** | | **Median** | **1SD** | | **2SD** | | |
| 1 | 6.08 | 7.56 | | 9.05 | | | 10.55 | | 12.06 | 5.66 | 7.13 | | 8.84 | | | 10.80 | | 13.04 |  |
| 2 | 7.58 | 9.49 | | 11.42 | | | 13.36 | | 15.32 | 7.52 | 9.37 | | 11.54 | | | 14.06 | | 16.96 |  |
| 3 | 8.90 | 11.21 | | 13.56 | | | 15.92 | | 18.30 | 9.28 | 11.46 | | 14.03 | | | 17.05 | | 20.58 |  |
| 4 | 10.19 | 12.92 | | 15.68 | | | 18.48 | | 21.30 | 10.92 | 13.39 | | 16.33 | | | 19.81 | | 23.92 |  |
| 5 | 11.45 | 14.60 | | 17.81 | | | 21.07 | | 24.37 | 12.45 | 15.19 | | 18.47 | | | 22.41 | | 27.11 |  |
| 6 | 12.73 | 16.34 | | 20.04 | | | 23.79 | | 27.61 | 13.93 | 16.94 | | 20.59 | | | 25.02 | | 30.37 |  |
| 7 | 14.07 | 18.20 | | 22.43 | | | 26.74 | | 31.11 | 15.39 | 18.70 | | 22.75 | | | 27.71 | | 33.80 |  |
| 8 | 15.44 | 20.14 | | 24.94 | | | 29.84 | | 34.82 | 16.86 | 20.49 | | 24.98 | | | 30.54 | | 37.45 |  |
| 9 | 16.88 | 22.20 | | 27.65 | | | 33.19 | | 38.81 | 18.36 | 22.34 | | 27.30 | | | 33.51 | | 41.32 |  |
| 10 | 18.50 | 24.49 | | 30.63 | | | 36.90 | | 43.26 | 19.88 | 24.24 | | 29.71 | | | 36.61 | | 45.35 |  |
| 11 | 20.34 | 27.00 | | 33.86 | | | 40.91 | | 48.12 | 21.41 | 26.19 | | 32.18 | | | 39.76 | | 49.40 |  |
| 12 | 22.37 | 29.65 | | 37.25 | | | 45.11 | | 53.21 | 22.89 | 28.07 | | 34.58 | | | 42.77 | | 53.14 |  |
| 13 | 24.53 | 32.40 | | 40.71 | | | 49.38 | | 58.39 | 24.21 | 29.77 | | 36.70 | | | 45.36 | | 56.19 |  |
| 14 | 26.83 | 35.25 | | 44.22 | | | 53.70 | | 63.62 | 25.25 | 31.12 | | 38.36 | | | 47.27 | | 58.24 |  |
| 15 | 29.13 | 38.04 | | 47.65 | | | 57.90 | | 68.74 | 26.00 | 32.09 | | 39.49 | | | 48.46 | | 59.30 |  |
| 16 | 31.33 | 40.67 | | 50.85 | | | 61.83 | | 73.55 | 26.51 | 32.72 | | 40.18 | | | 49.10 | | 59.74 |  |
| 17 | 33.38 | 43.11 | | 53.83 | | | 65.49 | | 78.06 | 26.86 | 33.09 | | 40.54 | | | 49.39 | | 59.86 |  |
| 18 | 35.33 | 45.43 | | 56.66 | | | 68.98 | | 82.37 | 27.10 | 33.34 | | 40.76 | | | 49.54 | | 59.88 |  |

**Supplementary table 2.** tPSMA-for-age (cm^2^) references for boys and girls. SD, standard deviation; tPSMA, total Paraspinal Muscle Area
