# Supplementary material for: Reference values for MRI‐derived psoas and paraspinal muscles and macroscopic fat infiltrations in paraspinal muscles in children
Source: J Cachexia Sarcopenia Muscle. 2022 Jul 19;13(5):2515–24. doi: 10.1002/jcsm.13049 (PMC9530503; doi:10.1002/jcsm.13049)
Supplement: Supplementary file 11 — Table S3. TMA‐for‐age (cm2) references for boys and girls. SD, standard deviation; TMA, Total Muscle Area [file JCSM-13-2515-s012.docx]

| **Age (years)** | **Boys** | | | | | | | | | **Girls** | | | | | | | | |  |
| --- | --- | --- | --- | --- | --- | --- | --- | --- | --- | --- | --- | --- | --- | --- | --- | --- | --- | --- | --- |
|  | **-2SD** | | **-1SD** | | **Median** | **1SD** | | **2SD** | | **-2SD** | | **-1SD** | | **Median** | **1SD** | | **2SD** | | |
| 1 | 10.51 | 12.61 | | 14.71 | | | 16.83 | | 18.94 | 9.51 | 11.85 | | 14.55 | | | 17.64 | | 21.14 |  |
| 2 | 12.97 | 15.61 | | 18.31 | | | 21.07 | | 23.88 | 12.31 | 15.19 | | 18.53 | | | 22.38 | | 26.79 |  |
| 3 | 15.,8 | 18.19 | | 21.44 | | | 24.83 | | 28.34 | 15.00 | 18.35 | | 22.56 | | | 26.80 | | 32.03 |  |
| 4 | 17.19 | 20.74 | | 24.54 | | | 28.59 | | 32.89 | 17.59 | 21.37 | | 25.82 | | | 31.08 | | 37.07 |  |
| 5 | 19.38 | 23.36 | | 27.74 | | | 32.51 | | 37.68 | 20.07 | 24.28 | | 29.26 | | | 35.12 | | 42.00 |  |
| 6 | 21.70 | 26.16 | | 31.16 | | | 36.73 | | 42.91 | 22.45 | 27.08 | | 32.60 | | | 39.15 | | 46.91 |  |
| 7 | 24.23 | 29.22 | | 34.93 | | | 41.40 | | 48.71 | 24.74 | 29.82 | | 35.89 | | | 43.15 | | 51.81 |  |
| 8 | 26.90 | 32.52 | | 39.03 | | | 46.52 | | 55.10 | 27.06 | 32.62 | | 39.30 | | | 47.32 | | 56.93 |  |
| 9 | 29.75 | 36.14 | | 43.60 | | | 52.27 | | 62.26 | 29.52 | 35.63 | | 42.98 | | | 51.83 | | 62.49 |  |
| 10 | 32.82 | 40.16 | | 48.77 | | | 58.80 | | 70.43 | 32.17 | 38.89 | | 47.00 | | | 56.79 | | 68.58 |  |
| 11 | 36.03 | 44.49 | | 54.42 | | | 66.00 | | 79.43 | 34.92 | 42.32 | | 51.25 | | | 62.02 | | 75.01 |  |
| 12 | 39.32 | 49.04 | | 60.45 | | | 73.73 | | 89.07 | 37.61 | 45.68 | | 55.42 | | | 67.16 | | 81.29 |  |
| 13 | 42.71 | 53.80 | | 66.79 | | | 81.87 | | 99.23 | 40.00 | 48.69 | | 59.15 | | | 71.73 | | 86.34 |  |
| 14 | 46.33 | 58.78 | | 73.40 | | | 90.40 | | 109.98 | 41.96 | 51.12 | | 62.12 | | | 75.33 | | 91.14 |  |
| 15 | 50.02 | 63.73 | | 79.95 | | | 98.93 | | 120.93 | 43.43 | 52.89 | | 64.24 | | | 77.83 | | 94.07 |  |
| 16 | 53.60 | 68.45 | | 86.20 | | | 107.17 | | 131.75 | 44.52 | 54.09 | | 65.59 | | | 79.41 | | 95.98 |  |
| 17 | 57.00 | 72.89 | | 92.08 | | | 115.06 | | 142.34 | 46.39 | 54.91 | | 66.44 | | | 80.42 | | 97.36 |  |
| 18 | 60.28 | 77.13 | | 97.74 | | | 122.75 | | 152.91 | 46.13 | 55.54 | | 67.05 | | | 81.19 | | 98.59 |  |

**Supplementary table 3.** TMA-for-age (cm^2^) references for boys and girls. SD, standard deviation; TMA, Total Muscle Area
