# Supplementary material for: Reference values for MRI‐derived psoas and paraspinal muscles and macroscopic fat infiltrations in paraspinal muscles in children
Source: J Cachexia Sarcopenia Muscle. 2022 Jul 19;13(5):2515–24. doi: 10.1002/jcsm.13049 (PMC9530503; doi:10.1002/jcsm.13049)
Supplement: Supplementary file 12 — Table S4. tMFI‐for‐age (cm2) references for boys and girls. SD, standard deviation; tMFI, total Macroscopic Fat Infiltrations in paraspinal muscles [file JCSM-13-2515-s005.docx]

| **Age (years)** | **Boys** | | | | | | | | | **Girls** | | | | | | | | |  |
| --- | --- | --- | --- | --- | --- | --- | --- | --- | --- | --- | --- | --- | --- | --- | --- | --- | --- | --- | --- |
|  | **-2SD** | | **-1SD** | | **Median** | **1SD** | | **2SD** | | **-2SD** | | **-1SD** | | **Median** | **1SD** | | **2SD** | | |
| 1 | 0.26 | 0.42 | | 0.66 | | | 1.01 | | 1.50 | 0.25 | 0.40 | | 0.60 | | | 0.87 | | 1.22 |  |
| 2 | 0.27 | 0.43 | | 0.67 | | | 1.02 | | 1.53 | 0.26 | 0.42 | | 0.64 | | | 0.94 | | 1.34 |  |
| 3 | 0.28 | 0.44 | | 0.67 | | | 1.03 | | 1.55 | 0.28 | 0.45 | | 0.70 | | | 1.04 | | 1.51 |  |
| 4 | 0.29 | 0.44 | | 0.68 | | | 1.03 | | 1.57 | 0.30 | 0.50 | | 0.78 | | | 1.19 | | 1.75 |  |
| 5 | 0.30 | 0.46 | | 0.70 | | | 1.08 | | 1.65 | 0.32 | 0.53 | | .086 | | | 1.32 | | 1.98 |  |
| 6 | 0.33 | 0.50 | | 0.77 | | | 1.17 | | 1.82 | 0.33 | 0.55 | | 0.90 | | | 1.42 | | 2.18 |  |
| 7 | 0.37 | 0.55 | | 0.84 | | | 1.30 | | 2.03 | 0.34 | 0.57 | | 0.94 | | | 1.52 | | 2.39 |  |
| 8 | 0.39 | 0.59 | | 0.91 | | | 1.42 | | 2.24 | 0.35 | 0.40 | | 1.00 | | | 1.64 | | 2.66 |  |
| 9 | 0.41 | 0.62 | | 0.97 | | | 1.52 | | 2.42 | 0.38 | 0.64 | | 1.08 | | | 1.82 | | 3.03 |  |
| 10 | 0.42 | 0.65 | | 1.02 | | | 1.63 | | 2.63 | 0.42 | 0.70 | | 1.19 | | | 2.03 | | 3.47 |  |
| 11 | 0.43 | 0.68 | | 1.09 | | | 1.76 | | 2.89 | 0.46 | 0.77 | | 1.31 | | | 2.26 | | 3.95 |  |
| 12 | 0.44 | 0.72 | | 1.17 | | | 1.92 | | 3.17 | 0.50 | 0.84 | | 1.44 | | | 2.49 | | 4.39 |  |
| 13 | 0.45 | 0.74 | | 1.24 | | | 2.05 | | 3.43 | 0.55 | 0.92 | | 1.57 | | | 2.72 | | 4.76 |  |
| 14 | 0.45 | 0.76 | | 1.29 | | | 2.17 | | 3.64 | 0.61 | 1.02 | | 1.72 | | | 2.93 | | 5.04 |  |
| 15 | 0.44 | 0.77 | | 1.32 | | | 2.25 | | 3.80 | 0.67 | 1.12 | | 1.88 | | | 3.13 | | 5.23 |  |
| 16 | 0.44 | 0.78 | | 1.36 | | | 2.34 | | 3.96 | 0.74 | 1.24 | | 2.05 | | | 3.33 | | 5.38 |  |
| 17 | 0.44 | 0.79 | | 1.40 | | | 2.43 | | 4.12 | 0.83 | 1.37 | | 2.22 | | | 3.53 | | 5.50 |  |
| 18 | 0.43 | 0.80 | | 1.44 | | | 2.52 | | 4.29 | 0.92 | 1.51 | | 2.40 | | | 3.71 | | 5.60 |  |

**Supplementary table 4.** tMFI-for-age (cm^2^) references for boys and girls. SD, standard deviation; tMFI, total Macroscopic Fat Infiltrations in paraspinal muscles
